# 

**Published:** 1888-08

**Authors:** 


### CONTENTS.

ORIGINAL COMMUNICATIONS:

Surgical Cases. By W. Z. Holliday, M. D., Harlem, Ga. .......... 321
A Case in Surgery. By R. H. Taylor, M. D., Griffin, Ga. .......... 327
Practical Experience in Typhoid Fever as it Occurs in Alabama. By Frank Prince, M. D., Bessemer, Ala. .......... 331
The External Application of Sulphur in Sciatic Neuralgia. By J. W. Cowden, M. D., Rock Island, Ill. .......... 340

SELECTIONS:

The Embryo Physician as a Specialist. By I. H. Nott, M. D., Goliad, Texas .......... 343

SOCIETY REPORTS:

Chicago Pathological Society .......... 350
The Clinical Society of Maryland .......... 355

CORRESPONDENCE:

Our New York Letter .......... 361
The Louisiana Medical Society *versus* the Editorial Staff of the New Orleans Medical and Surgical Journal .......... 364

BOOK REVIEWS:

Cyclopedia of Obstetrics and Gynecology. Grandin .......... 372
On a New Treatment of Chronic Metritis, and Especially of Endometritis, with Intra-Uterine Chemical Galvano-Cauterizations. Apostoli .......... 373
The Pathology, Diagnosis and Treatment of the Diseases of Women. Hewitt .......... 374

EDITORIAL:

Salfonal—A New Hypnotic .......... 375

Adulteration of Drugs .......... 376

A City Hospital for Atlanta .......... 376

Counter-Prescribing .......... 377

Items .......... 378

